# Magnitude and correlates of caesarean section in urban and rural areas: A multivariate study in Vietnam

**DOI:** 10.1371/journal.pone.0213129

**Published:** 2019-07-26

**Authors:** Myriam de Loenzien, Clémence Schantz, Bich Ngoc Luu, Alexandre Dumont

**Affiliations:** 1 Centre Population et Développement, Institut de Recherche pour le Développement, Université Paris Descartes, Inserm, France; 2 Institute for Population and Social Studies, National Economic University, Hanoi, Vietnam; National Cancer Centre, JAPAN

## Abstract

Caesarean section (CS) can prevent maternal and neonatal mortality and morbidity. However, it involves risks and high costs that can be a burden, especially in low and middle income countries. The aim of this study is to assess its magnitude and correlates among women of reproductive age in the urban and rural areas of Vietnam. We analyzed microdata from the national Multiple Indicator Cluster Survey (MICS) conducted in 2014 by using a representative sample of households at the national level in both urban and rural areas. A total of 1,350 women who delivered in institutional settings in the two years preceding the survey were included. Frequency and percentage distributions of the variables were performed. Bivariate and multivariate logistic regression analyses were undertaken to identify the factors associated with CS. Odds ratios with a 95% confidence interval were used to ascertain the direction and strength of the associations. The overall CS rate among the women who delivered in healthcare facilities in Vietnam has rapidly increased and reached a high level (29.2%). After controlling for significant characteristics, living in urban areas doubles the likelihood of undergoing a CS (OR = 1.98; 95% CI 1.48 to 2.67). Maternal age at delivery over 35 years is a major positive correlate of CS. Beyond this common phenomenon, different distinct lines of socioeconomic and demographic cleavage operate in urban compared with rural areas. The differences regarding the correlates of CS according to the place of residence suggest that specific measures should be taken in each setting to allow women to access childbirth services that are appropriate to their needs.

## Introduction

Caesarean section (CS) can prevent maternal and neonatal mortality and morbidity when medically justified. However, it involves risks and high costs that can be a burden, especially in low and middle income countries [1] [2]. There is no consensus on an optimal population-level frequency of CS. However, a global concern around CS rates has developed since a large part of CS is considered to be not medically justified [3] [4]. Urbanization is related not only to a population that moves from a rural to an urban area and an increased concentration of people who live in urban areas but also to the entire process of societal adaptation to the subsequent changes, and urbanization has been identified as a prominent contributing factor to CS practices in several countries and areas [5] [6] [7] [8] [9] [10] [11]. However, this influence is controversial [12] [13] [14].

Vietnam, which transformed from a low- to a middle-income country in the last decade, has witnessed increasing CS rates concomitantly with urbanization. The proportion of women who undergo CS has been multiplied by almost 7 within a 17-year period, from 3.4% in 1997 to 27.5% in 2013–14 [15] [16]. This percentage is among the highest in the region [17] [18]. This rising trend that shows no sign of abatement is occurring in a context of rapid socioeconomic and demographic changes marked with increasing urbanization. Urbanization accelerated in the 1990s [19] following the reforms in the mid-1980s from a centralized system to a market-oriented economy under state guidance [20]. The country has shifted its policy from the promotion of intermediate-level cities in the 1990s-2000s to more investments in large metropolitan areas that aimed to act as drivers of the economy [21]. The proportion of people who live in urban areas rapidly increased from 19.2% in the 1980s to 23.7% in 1999 and 34.0% in 2015 [22] [23].

Simultaneously, rural-urban inequalities have decreased. In particular, CS rates have increased at a higher pace in rural areas, where they have multiplied by 9 (from 2.3% to 21.0%), than in urban areas, where they have multiplied by 3 (from 13.6% to 43.3%). Consequently, the urban-rural ratio of the proportion of women who underwent CS dropped (from 5.9 in 1997 to 2.1 in 2014) [16] [24].

We propose to measure the influence of living in urban compared with rural areas on childbirth practices and to explore the possible pathways of the influence of the place of residence on CS in Vietnam. By using microdata from a nationally representative sample, we provide the demographic and socioeconomic profiles associated with high CS rates. Subsequently, we present the correlates of the CS rates by distinguishing between women who live in rural and urban areas. Our main argument is that beyond the apparent overall convergence, CS practices diverge between rural and urban areas not only in their magnitude but also in their dynamics.

## Literature review

For several decades, living in urban areas in low- and middle-income countries in Asia, Africa and Latin America has been associated with higher CS rates [5] [6], even after controlling for multiple factors [7] [8] [9] [10] [11]. In many cases, this involves CS deliveries for nonabsolute medical indications [25]. However, the relationship between high CS rates and urbanization appears to be nonsignificant in various settings [12] [13]. Further analyses that consider the level of urbanization complement these results. A study that use data from 29 countries in Asia, Africa and Latin America showed higher CS rates in urban areas than in periurban areas [26] whereas the reverse prevailed in Cambodia [27].

The drivers of CS overuse fall into the three following interconnected categories: healthcare systems, financial reimbursement, organizational design and cultures; health professionals; and childbearing women, families, communities and the broader society [4]. In Vietnam, the increase in CS rates has occurred in the context of a marked development of childbirth biomedicalization fostered by rapid economic growth and recent investment in district hospitals. Only 71% of pregnancies were followed up by a doctor, nurse or midwife in 1997, whereas almost all pregnancies were followed up by a doctor, nurse of midwife in 2014 (95.8%) [16] [24] [28]. The number of prenatal care visits has dramatically increased. Although very few women attended 4 visits or more in 1997 (4.2%), this number was much higher 17 years later (73.7%) [24] [29]. Previous research in other low- and middle-income countries show the positive influence of the private healthcare sector [11] [30] [31]. In Vietnam, the development of private healthcare facilities has undergone a major evolution following the Doi Moi reforms launched in the mid-1980s. The highest rates of CS are observed among the richest household quintiles in many low- and middle-income countries [6] [9] [32]. In turn, most richer rural households had higher CS rates than poorer rural households had [33]. Both trends are observed in Vietnam [33]. The positive link between health insurance and CS practice observed in neighboring China [34] may also apply. The trends towards lower fertility in urban areas favor higher levels of antenatal care attendance and CS use [35]. This may hold for Vietnam, as this country has ended its demographic transition. Among the other factors of a reduction in fertility, a high age at delivery has been reported as associated with high CS rates in Vietnam [36] [37] and other countries [10] [11] [13] [35]. A study performed in Vietnam showed that discussions with relatives also play a moderating role in helping women to avoid CS [12]. The wish to deliver at a propitious time is also a reason for a CS request, especially in countries that refer to the lunar calendar such as China and Vietnam [4] [38]. Other potential factors of CS include the characteristics of the child. Previous findings regarding periurban settings in Northern Vietnam showed no effect of the weight of the newborn on the mode of delivery [37]. In addition, in Northern Vietnam, the preference for a boy increases the recourse to CS [39].

## Materials and methods

### Data

We used microdata from the Multiple Indicator Clusters Survey (MICS) conducted from December 2013 to April 2014 by the General Statistical Office in collaboration with UNICEF. The urban and rural areas within each region were identified as the main sampling strata. The sample was selected in two stages: census enumeration areas were selected within each stratum, and households were selected within each enumeration area [16]. This dataset provided statistically representative samples of women aged 15–49 years at the national and regional levels and for each type of setting (rural compared with urban).

With this dataset, we focused on women who had a birth of a singleton at least once during the 2 years prior to the survey. Among these women, we considered those who delivered at an institutional setting. This choice allowed us to focus on the determinants of CS This focus did not significantly change the results compared with considering all deliveries including the deliveries that occurred at home, as women who delivered at an institutional setting involve almost all the population (94.4%). Only the last birth of each woman was considered to avoid counting a woman twice who had two single childbirths during the two years under study, which included very few women. This approach also has the advantage of reducing recall bias. Overall, the 1,350 women who participated in a face-to-face interview after completing individual questionnaires were included in this study.

### Outcome measures and covariates

The outcome variable for this study was the woman’s mode of delivery, either through CS or vaginal delivery. The main covariate for this study was the place of residence (urban compared with rural area). The influence of this variable was explored by controlling for other correlates.

Regarding biomedical characteristics, the correlates included the place of delivery (public compared with the private healthcare sector), the number of antenatal care (ANC) visits and the birth weight and size of the newborn as declared by the mother. Given that the 2016 Vietnamese guidelines recommend at least four ANC visits [40] and that the standard ANC model recommends at least eight ANC visits [41], we compared women who had less than 4, women who had 4 to 7 and women who had 8 ANC visits or more. The upper limit adopted for the child’s weight over average (3.6 kg and over) is based on Vietnam’s 2016 guidelines on Reproductive Healthcare which state that childbirth weight over 3.5 kg raises risks of a difficult delivery [40]. The lower limit (less than 2.5 kg) is the usual figure for underweight newborns [41].

Demographic characteristics included the maternal age at delivery (15 to 19 years, 20 to 34 years, and 35 years and over), the woman’s past experience of childbirth (primiparous compared with multiparous), and the sex of the newborn.

Socioeconomic characteristics which were analyzed as distal determinants included the highest level of education attended (primary or less, secondary, and tertiary including college and university), the quintile of the wealth of the household (poorest, poor, middle, rich or richest), the ethnicity of the household head (Kinh and Hoa ethnic groups compared with minority ethnic groups), and the region (North Central, Mekong River Delta, Red River Delta, Northern Midlands, Central Highlands or the Southeast).

### Analysis

We conducted a bivariate analysis and stepwise logistic regression to assess the characteristics associated with CS practice as opposed to vaginal delivery. The bivariate analyses used the women’s sample weights. The multivariate logistic regression models are controlled for the cluster effect. They allowed for comparisons between models for all women and for only women who live in rural or urban areas. For each of these 3 groups, two models were tested. The first model, which is referred to as the restricted model, included only the biomedical and demographic variables, which are analyzed as proximate determinants. The second model, which referred to as the complete model, included socioeconomic characteristics in addition to all variables from the restricted model. For each model and for the chi-square tests, we draw on one level of risk (p < 0.05). We provide odds ratios (ORs) as a measure of the likelihood to undergo a CS associated with each characteristic and confidence interval (CI) of the ORs as a measure of the level of precision of this indicator. All statistical analyses were performed with IBM PASW Statistics 18 software at Paris Descartes University.

### Compliance with ethical standards

The Vietnam General Statistics Office (GSO) and the United Nations Children’s Fund (UNICEF) approved the tools of the Vietnam Multiple Indicator Cluster Survey (MICS) before the survey was conducted, according to the ethical standards in the 1964 Declaration of Helsinki and its later amendments or comparable ethical standards. Participation was voluntary, and informed consent was obtained from all the individual participants included in the study. The MICS data are freely available through the UNICEF MICS website, and there is no need to obtain ethical approval before using the data. To access data from the MICS website, a written request was submitted to UNICEF, and permission was granted.

## Results

The overall CS rate among the women who delivered in healthcare facilities was particularly high (29.2%). It was almost twice as high in urban (42.4%) than in rural areas (22.9%). Overall, almost one-third of the women lived in the urban areas (30.7%). Table 1 provides an overview of the social and demographic profiles of the women who delivered in healthcare facilities and the corresponding rates of CS.

**Table 1:** Social and demographic profile of the women who had a birth of a singleton in the two years preceding the survey in healthcare facilities and the corresponding rates of caesarean section (CS) (n = 1350).

		Distribution			CS rates			
		Urban	Rural		Urban		Rural	
		%	%	p	%	p	%	p
Place of delivery	Public healthcare sector	92.5	97.2	**	42.4	*	22.9	
	Private healthcare sector	7.5	2.8		61.3		11.5	
Antenatal care visits	3 or fewer	12.8	25.2	**	29.6	**	17.4	**
	4 to 7	38.9	50.9		34.8		22.9	
	8 or more	48.3	23.9		55.0		27.4	
Weight of the newborn	Less than 2.5 kg	3.9	4.6	**	50.0	*	35.7	**
	2.5 to 3.5 kg	78.6	85.1		41.1		21.1	
	Over 3.5 kg	17.6	10.3		54.8		29.9	
Size of the newborn	Average	76.1	79.8	**	39.9	**	22.7	
	Smaller than average	8.2	9.7		51.5		19.8	
	Larger than average	15.7	10.5		58.5		24.7	
Maternal age at delivery	15–19	5.3	8.9	**	22.7	**	17.1	**
	20–34	84.6	83.1		43.0		22.1	
	35–49	10.1	8.0		61.9		34.2	
Parity	Multiparous	57.1	56.8		42.4		20.5	*
	Primiparous	42.9	43.2		45.5		25.2	
Women’s education	Primary or less	9.9	14.3	**	31.7	**	23.1	
	Secondary	51.1	66.6		38.7		21.5	
	Tertiary	39.0	19.0		53.7		26.8	
Wealth quintile	Poorest	3.4	20.6	**	21.4	**	20.8	
	Poor	8.9	25.7		36.1		22.9	
	Middle	14.7	24.2		31.1		23.9	
	Rich	25.1	21.9		34.6		23.3	
	Richest	48.0	7.6		55.3		21.1	
Ethnicity	Kinh and Hoa	93.5	85.8	**	45.1	*	23.6	
	Ethnic minorities	6.5	14.2		25.9		17.3	
Region	North Central	20.2	22.0	**	51.2		26.2	*
	Mekong R. Delta	12.0	19.6		52.0		21.3	
	Red River Delta	23.1	25.5		44.8		17.6	
	Northern Midlands	9.2	14.3		33.3		26.9	
	Central Highlands	5.5	6.9		26.1		15.4	
	Southeast	29.9	11.6		42.7		28.4	
Number of women		415	935		415		935	

Abbreviations and signs: CS, cesarean section; OR, odds ratio; CI, confidence interval;

**, p ≤ 0.05;

*, p ≤ 0.10

Data are from the 2014 Vietnam Multiple Indicator Cluster Survey (MICS)

The results confirm that the urban context was particularly favorable to CS. Two main correlates linked to higher levels of CS were more prevalent in the urban areas than in the rural areas. First, CS rates were much higher among women who had at least 8 antenatal care visits than among women who had 7 ANC visits or less. Furthermore, the proportion of women who had at least 8 antenatal care visits had more than doubled in the urban areas compared with the rural areas. Second, compared with the CS rates for women aged 15–19 years, the CS rate had at least doubled for women who deliver after age 35 years. This age gap was wider in the urban areas than in rural areas. Furthermore, the maternal age at delivery was lower in the rural areas than in the urban areas.

Two indicators associated with a higher level of CS in urban areas only showed more favorable socioeconomic situations for women who live in urban areas. First, a much larger proportion of women reached a tertiary level of education in urban areas (39.0%) than in rural areas (19.0%). Additionally, in urban areas, the CS rates were much higher among women who had reached a tertiary level of education (53.7%) than among women who had a primary level of education or less (31.7%). Second, the proportion of women who live in the richest household quintile reached a much higher level of CS use in urban areas (48.0%) than in rural areas (7.6%) and in the urban areas, the proportion of women recurring to CS is much higher among women who live in the richest households (55.3%) than among women who live in the poorest households (21.4%).

A higher number of antenatal care visits, a higher maternal age at delivery, higher levels of education, and wealth, in urban areas combined with the higher CS rates among people in these groups help us to understand part of the remaining urban rural gap regarding CS rates. However, a more in-depth analysis is needed to document the relative influence of each of these correlates, which is achieved by using a multivariate analysis. The results of the analysis of the correlates of CS for the entire population are displayed in Table 2.

**Table 2:** Multivariate analysis of the factors associated with caesarean delivery (n = 1350).

	Restricted model			Complete model		
	OR	95% CI		OR	95% CI	
Place of residence (reference = rural)						
Urban	2.25**	1.74	2.90	1.98**	1.48	2.67
Place of delivery (reference = public sector)						
Private sector	1.34	0.81	2.20	1.27	0.75	2.15
Antenatal care visits (reference = 4 to 7)						
3 and less	0.78	0.54	1.12	0.83	0.56	1.23
8 or more	1.49**	1.11	2.00	1.49**	1.08	2.05
Weight of newborn (reference = 2.5 to 3.5 kg)						
Less than 2.5 kg	1.96**	1.09	3.53	2.16**	1.18	3.95
Over 3.5 kg	1.61**	1.11	2.34	1.56**	1.07	2.28
Maternal age at delivery (reference = 20–34)						
15–19	0.58	0.32	1.03	0.67	0.37	1.21
35–49	2.09**	1.40	3.14	2.18**	1.44	3.31
Parity (reference = multiparous)						
Primiparous	1.42**	1.07	1.89	1.39**	1.04	1.86
Women’s education (reference = secondary)						
Primary or less				1.06	0.68	1.65
Tertiary				1.28	0.91	1.80
Household wealth quintile (reference = Poorest)						
Poor				1.14	0.66	1.96
Middle				1.16	0.74	1.83
Rich				0.90	0.57	1.40
Richest				1.43	0.90	2.27
Ethnicity (reference = Kinh and Hoa)						
Ethnic minorities				0.62**	0.41	0.96
Region (reference = North Central)						
Mekong River Delta				0.79	0.52	1.22
Red River Delta				0.59**	0.38	0.91
Northern Midlands				1.06	0.71	1.57
Central Highlands				0.52	0.33	1.09
Southeast				0.78	0.51	1.20

Abbreviations and signs: CS, cesarean section; OR, odds ratio; CI, confidence interval;

**, p ≤ 0.05;

*, p ≤ 0.10

Data are from the 2014 Vietnam Multiple Indicator Cluster Survey (MICS)

We first examined a model that considered only the demographic and medical characteristics and that focus on the intermediate variables. The results show that after controlling for the significant characteristics, living in urban areas more than doubled the likelihood of undergoing a CS (OR = 2.25; 95% CI 1.74 to 2.90, see the restricted model). In addition to the place of residence, delivering at 35 years of age or over strongly favored undergoing CS. Conversely, the likelihood of having CS was halved for women aged 15–19 years compared with women aged 20–34 years. The weight of the newborn was also associated with high CS rates, for overweight and even more strongly for underweight newborns. A high number of antenatal care visits increased the likelihood of undergoing CS. Furthermore, primiparous women tended to use CS more than women who have delivered before.

These trends remained the same when the socioeconomic variables were included. However, most of their effects had weakened. Urbanization nevertheless remained strongly associated with CS, and it almost doubled the likelihood of using this delivery mode (OR = 1.98; 95% CI 1.48 to 2.67). Conversely, the complete model shows that women belonged to a household headed by a member of a minority ethnic group had a much lower likelihood to have CS than women who belonged to a household headed by a person from the Kinh or Hoa ethnic groups. This likelihood also decreased for women who lived in the Red River Delta.

To better understand the contrasts between the urban and rural dynamics and the correlates of CS, we separately studied women from each place of residence. The results are displayed in Table 3. The results show two models, namely, one model concerning urban areas, and the other model concerning rural areas.

**Table 3:** Odds of undergoing caesarean section (CS) for all women according to their place of residence: Urban (n = 415) and rural (n = 935) areas.

	Urban						Rural					
	Restricted model			Complete model			Restricted model			Complete model		
	OR	95% CI		OR	95% CI		OR	95% CI		OR	95% CI	
Place of delivery (reference = public sector)												
Private sector	2.39**	1.21	4.75	1.92	0.91	4.06	0.53	0.16	1.79	0.48	0.14	1.67
Antenatal care visits (reference = 4 to 7 visits)												
3 or less	0.87	0.49	1.54	0.99	0.51	1.92	1.24	0.83	1.83	0.73	0.45	1.17
8 visits or more	2.11**	1.38	3.23	2.04**	1.29	3.24				1.20	0.75	1.94
Weight of newborn (reference = 2.5 to 3.5 kg)												
Less than 2.5 kg	1.55	0.62	3.88	1.82	0.64	5.19	2.03	0.98	4.20	2.15**	1.01	4.55
Over 3.5 kg	1.77**	1.08	2.89	1.60	0.98	2.63	1.60	0.91	2.80	1.54	0.88	2.71
Maternal age at delivery (reference = 20–34)												
15–19	0.45	0.17	1.20	0.56	0.19	1.67	1.59	0.30	1.17	0.69	0.34	1.41
35–49	2.49**	1.37	4.55	2.76**	1.45	5.25	1.98**	1.13	3.45	1.97**	1.10	3.50
Parity (reference = multiparous)												
Primiparous	1.30	0.89	1.91	1.30	0.87	1.93	1.55**	1.04	2.32	1.43	0.93	2.20
Women’s education (reference = secondary)												
Primary or less				0.94	0.46	1.92				1.17	0.67	2.05
Tertiary				1.36	0.84	2.19				1.24	0.75	2.06
Household wealth quintile (reference = middle)												
Poorest				0.91	0.26	3.22				1.03	0.56	1.89
Poor				1.40	0.59	3.31				1.02	0.61	1.72
Rich				1.17	0.59	2.31				0.83	0.47	1.48
Richest				1.99**	1.01	3.95				0.80	0.35	1.79
Ethnicity (reference = Kinh and Hoa)												
Ethnic minorities				0.80	0.34	1.89				0.57**	0.34	0.94
Region (reference = North Central)												
Mekong River Delta				1.07	0.55	2.05				0.69	0.38	1.22
Red River Delta				0.60	0.31	1.15				0.56	0.31	1.02
Northern Midlands				0.52	0.26	1.07				1.31	0.81	2.12
Central Highlands				0.36**	0.17	0.74				0.65	0.37	1.16
Southeast				0.51**	0.28	0.93				1.00	0.55	1.83

Abbreviations and signs: CS, cesarean section; OR, odds ratio; CI, confidence interval;

**, p ≤ 0.05

Data are from the 2014 Vietnam Multiple Indicator Cluster Survey (MICS)

In both models, the maternal age at delivery of over 35 years was a major positive correlate of CS. Beyond this common phenomenon, distinct lines of socioeconomic and demographic cleavage operate in urban compared with rural areas.

Two factors had a positive influence on CS for women who live in urban areas, without having a significant effect on women who live in rural areas. In urban areas, women were more than twice as likely to undergo CS when they had at least 8 antenatal care visits and this effect remained even when controlled by socioeconomic characteristics. Women were also more than twice as likely to have a CS when they delivered in the private healthcare sector. However, this effect disappeared when socioeconomic factors were included.

The newborn’s weight was specific as it differed between the rural and the urban areas. In urban areas, women whose child’s weight was over average tended to undergo CS whereas in rural areas, women whose child’s weight was below average tended to have a CS.

Finally, the influence of socioeconomic characteristics also differed according to the place of residence. In urban areas, CS occurred twice more frequently among women who belong to the richest quintile of household wealth than among women who belong to the median level. CS was also twice as less prevalent in the Central Highlands and the Southeast than in the North Central region. In contrast, in the rural areas, parity had a significant effect in the restricted model: primiparous women were twice more likely to undergo CS than multiparous women. However, this effect did not remain when socioeconomic factors were included. In the complete model, the odds of undergoing CS were almost halved for women who belong to minority ethnic groups.

## Discussion

The overall CS rate among the women who delivered in healthcare facilities in Vietnam in 2014 was particularly high (29.2%) compared with many low- and middle-income countries [5] [6].

Despite the narrowing rural urban gap, which has also been observed regarding more generally childbirth medicalization in this country [16] [24], the findings of our study confirm our primary assumption that the place of residence had a significant effect on CS practices in Vietnam in 2013–14. These findings update the previous results that show a nonsignificant influence of urbanization on CS in the early 2000s [12]. Women who live in urban areas are twice as likely to deliver by CS than women who live in rural areas. At the same time, despite growing levels of urbanization, nearly half of all CSs still occur in rural areas, which has been the case for the last two decades [15] [24] [42]. This trend can be explained by the combination of doubling urbanization rates since 1997 and a more rapid increase of CS rates in rural areas than in urban areas.

In this study, we consider that the decision to undergo CS results from a negotiation between the caregiver and the patient and is established by proximate determinants. Among them, biomedical and demographic determinants play a major role. They concern the characteristics linked to biomedical procedures such as antenatal care, place of delivery, characteristics of the newborn, parity and maternal age at delivery. These proximate determinants are in turn verified by more distal determinants, such as social, cultural and political characteristics at the individual, interindividual and collective levels. They include not only women’s human, economic and social capital but also cultural beliefs, values and norms regarding family and gender relations, interactions between social groups, the media and formal institutions, the welfare state and national policies and economic conditions. Due to contrasted modes of socialization and levels of equipment, underlying processes related to these phenomena differ between rural and urban areas.

Our study reveals that a maternal age of over 35 years at childbirth more than doubles the likelihood of undergoing a CS and that this effect is stronger in urban areas, where childbearing is experienced slightly later than in rural areas. This difference reveals contrasting perceptions regarding the relationship between motherhood and age. Information and communication targeted at women and healthcare workers may help them to strengthen women’s confidence in their ability to deliver vaginally even when they are aged 35 years and over. This information could be conveyed during antenatal care visits which have already reached a high number in this country.

Indeed, almost half of women have at least 8 antenatal care visits, which is the number required by international standards [41]. However, the positive effect of a high number of antenatal care visits on CS rates that prevails in urban areas as our study has shown, suggests that these visits marked by the intensive use of antenatal ultrasound technology [43] raise anxiety which in turn fosters CS use as witnessed in eastern China [44]. Consequently, the content of these visits should focus less on the use of high-level technology and more on social and psychological support, communication and training to improve the preparation for vaginal delivery.

This intensive use of high-level technology particularly concerns particularly the private healthcare sector, which is strongly involved in pregnancy follow-up [43]. In contrast with previous research that shows the clear influence of the private healthcare sector on CS use in other low- and middle-income countries [11] [31], our study indicates that the private healthcare sector has a positive and strong influence on CS use but only in urban areas. This influence is explained by socioeconomic factors, for example the high level of recourse to CS by women who live in the richest household quintile. Midwifery service provisions in the private healthcare sector are scarce, especially in rural areas [28]. These provisions’ role in urban areas may be underestimated due to the offering of private services in public health facilities. A more in-depth investigation that distinguishes between private and public services within the public healthcare sector could provide more insights. Further explorations of our data show that the proportion of women who deliver in the private healthcare sector varies widely across regions. For example, the proportion is close to zero in the Northern Midlands and Mountain areas but reaches 20% in the urban Mekong River Delta. The highest rates of CS observed among the richest household quintiles illustrates the persistence of inequalities in Vietnam despite some progress [45] [46]. This suggests that much work remains to reduce socioeconomic inequalities. One of these measures that is underway is the generalization of social insurance coverage, which, despite problems with low protection levels, especially in rural areas [47], covers 70% of the population [48].

The weight of the newborn over average significantly increases the likelihood of undergoing a CS in urban areas, whereas it has no significant effect in rural areas. Conversely, CS is significantly linked to low birthweight in rural areas. Given that the upper limit of 3.5 kg that is used in Vietnam’s guidelines is low compared with international standards [41], discussion at the highest political levels and audits at medical healthcare infrastructures could help examine the possibility to adopt a less strict limit. Such a change could allow women with fetuses around 3.5 kg, especially in urban areas, to have a greater chance of avoiding non-medically indicated CS.

Interestingly, women’s education level has no significant effect. Women’s abilities to argue their case and seek legal recourse in case of medical complications may act as a more powerful form of pressure on health staff in urban areas than in rural areas [6] [30] [49]. In urban areas, a higher level of exposure to the media and women’s greater involvement in formal professional activities could make it more difficult for health personnel to resist women’s requests to undergo CS. The media hold power over healthcare facilities through the diffusion of information on their practices and results. Part of this power is used through social networks, which shape public opinion. In contrast, in rural areas where the family size is larger, women may benefit from closer links to their relatives [29].

CS rates vary according to the region in urban areas but not in rural areas where the key factor is ethnicity, which in turn is not relevant in urban areas. Women from minority ethnic groups are less likely to perform a CS regardless of the other characteristics considered in our study. This gap is widened by a lower level of birth in health facilities and a lower level of assistance by skilled attendants during delivery among women who belong to minority ethnic groups [28]. It argues in favor of pregnancy follow-up and delivery monitoring which should be designed and organized to consider differences in attitudes and behaviors towards childbirth, which may involve interpersonal communication and transmission.

Our results also suggest that in rural areas, the higher level of CS among primiparous women may be partly explained by the fact that they belong to the Kinh and Hoa ethnic group, where fertility reaches lower levels. CS determinants combine with one another. One heuristic concept that can integrate the factors of CS may be “urban liveability”, which encompasses not only the physical setting but also social interactions and has been studied in relation to the social determinants of health in northern countries [50].

The different CS rates between rural and urban areas may also be explained by different levels of healthcare equipment. In Vietnam, where the health system is pyramidal with a special status for main cities [51], the two metropolitan areas of Hanoi in the Red River Delta and Ho Chi Minh City in the Southeast region play key roles. The fact that more than half of the urban population lives in either the Southeast region (29.9%) or the Red River Delta (23.1%) reveals the demographic weight of these two main metropoles. At the other extreme, almost half of the women who live in rural areas reside in the Red River Delta (25.5%) or the North Central region (22.0%).

The two main metropolitan areas in the country benefit from a concentration of highly equipped healthcare facilities in densely populated zones served by viable transportation and road networks. This situation leads to a high number of deliveries within specialized healthcare services. The rural–urban divide is further strengthened by the competition between health infrastructures following the “autonomization” policy launched in the 2000s, which spurs hospitals to make profits from investments [52]. In urban areas where health personnel are more heavily subject to time pressures and overcrowded services, CS enables more predictable staff management and shortens the delivery duration. Public hospitals at the tertiary level are closely monitored [51]. These hospitals where CSs are performed play a pioneering role in the elaboration and implementation of health policies at the national level.

This study has limitations. First, we do not know the reason why the CS deliveries under study were performed. In particular, we cannot distinguish between medically indicated CS deliveries from CSs performed based on the patient’s request. Therefore, we can only uncover general trends. Second, we do not distinguish between the several levels or types of urbanization; this type of analysis would require a large sample size. Third, the place of residence may not coincide with the place of delivery. Therefore, we capture the impact of the long-term influences of the context rather than the impact of possible adaptation through migration. Fourth, our statistical analysis provides indications of correlations rather than causal links. However, we are convinced that this study provides useful insights into the influence of urbanization on CS by emphasizing its major determinants and suggesting a way to approach this complex phenomenon with existing data representative of the national level.

## Conclusion

The overall CS rate among the women who delivered in healthcare facilities in Vietnam is particularly high (29.2%). Our paper aims to update the general trends regarding this phenomenon and better understand the correlates of the differences between rural and urban areas. Our results show that after controlling for significant characteristics, living in urban areas more than doubles the likelihood of undergoing a CS. Maternal age at delivery over 35 years is also a major positive correlate of CS. Beyond this common phenomenon, contrasting models exist regarding the determinants of the recourse to high levels of CS between rural and urban areas. This contrast suggests that actions to reduce unnecessary caesarean deliveries should be adapted to each context. Accordingly, our results show the importance of considering not only medical and demographic factors but also socioeconomic determinants when designing programs to improve women’s childbirth conditions. For example, the case of ethnicity needs to be addressed. This approach involves policies at many different levels regarding the regulation of the healthcare sector, the training of healthcare providers and the sensitization of the entire population, with means that are appropriate to their conditions of living. Further research must be conducted to design such programs and to provide guidance on this complex issue.
